# Mind-Personality Relations: Comment on Demetriou et al., 2018

**DOI:** 10.3390/jintelligence6040052

**Published:** 2018-12-06

**Authors:** Phillip L. Ackerman

**Affiliations:** School of Psychology, Georgia Institute of Technology, 654 Cherry Street, Atlanta, GA 30332, USA; plackerman@gatech.edu; Tel.: +1-404-894-5611

The article by Demetriou et al. [1] represents an impressive documentation of the relations between intellectual ability constructs and personality constructs, in multi-wave studies of children and adolescents. The authors examined a variety of different measures of intellectual abilities, personality traits, and other variables (e.g., self-concept, emotional intelligence). They provide extensive and innovative analyses of the changes in both intellectual abilities and non-ability traits across the waves of data collection, and they also report some data on academic achievement criterion data. They also discuss several perspectives on both the structure of intellectual abilities and the structure of personality traits, though the main focus is on attempts to converge on the more “general” constructs (such as fluid intelligence, a construct they call “cognizance”, and a general factor of personality). Across these studies, the authors reach conclusions that intellectual ability changes during childhood and adolescence are related to personality constructs, such as “likeability”, though in many cases the effects are not consistent across the various ages under investigation.

The studies underlying the discussion are diverse, in terms of samples, underlying constructs being assessed, and measures used to assess the constructs. This could be considered to be a prominent weakness of the article, because it was not clear that one could reasonably ascertain that there was sufficient communality of measures to allow for generalization across the studies or beyond the studies. Similarly, one could argue about whether some of the measures were even suitable assessments of the target constructs (e.g., choice reaction tasks and a Stroop-based task as indicators of cognitive abilities, or forward digit span as an indicator of working memory ability). A variety of other concerns could be expressed about each of the study characteristics, such as the make-up of the self-report measures, and whether they were suitably consistent in content or meaning for children and adolescents of different ages. These concerns do limit the potential impact of the individual study results and the overall consistency of the conclusions that could be drawn from the investigations.

Nonetheless, my sense is that one should regard the work of these authors as an example of the “context of discovery” (attributed to Reichenbach—see [2]), as opposed to the “context of justification”. In the general formulation of these different contexts, the context of discovery concerns how investigators come to discoveries or hypotheses. There need not be exceptional consistency or even experimental rigor in how such ideas are formulated. The context of justification, however, requires exceptional rigor in evaluating whether such discoveries or hypotheses are confirmed or disconfirmed (falsification). The concerns expressed above regarding samples, underlying constructs being assessed and measures used to assess the constructs, pertain mostly to the context of justification.

From the perspective of the context of discovery, the Demetriou et al. article provides several interesting findings, relating to the authors’ speculations on the respective structures of intellectual abilities and non-ability traits, to the reported results on the nature and directions of influences of personality and other self-constructs on individual differences in intellectual growth. Such findings provide for numerous hypotheses that will, in turn, require extensive testing with common methods and cross-sample consistencies, prior to establishing whether these effects are sufficiently robust to be justified into an overarching representation of how personality and intellectual abilities influence one another during child and adolescent development. In particular, constructs of social likeability and “cognizance” appear to be especially promising for future inquiry, and conscientiousness remains a construct without a clear pattern of relations with either intellectual ability or academic performance measures.

At a conceptual level, one question strikes me as important. Numerous investigations, including the one currently under discussion, have documented associations (mostly correlational) between intellectual ability measures and non-ability measures (e.g., personality, self-concept, and other self-report measures). These relations are often found to be statistically significant, and though most such relations are modest in magnitude, some reach levels of moderate or higher associations. Yet, there have not been, to my knowledge, any theoretical proposals that specify *how related* these various constructs are expected to relate to one another. Conceptually, the value of this area of research will come from specification of the magnitude of such relations. For example, broad measures of intellectual ability (such as omnibus IQ tests) may account for as much as 30–40% of the individual-differences variance in academic performance, especially for children and early adolescents. However, it remains unclear how much variance in intellectual ability levels, or in changes to intelligence during development *should* be accounted for by personality trait and other non-ability measures. As discussed elsewhere [3], although intelligence measures were specifically created to predict individual differences in academic performance, the same is not true of most personality and other non-ability trait measures. The question of “why” non-ability measures relate to intellectual development and academic performance can be answered, and the current article provides some potential insights. But the question of “how much should non-ability measures relate to intellectual development?” remains to be addressed.

One final observation bears noting here. Early investigations of the relations of personality and intellectual ability during development [4] focused more on *describing* developmental changes among members of a longitudinal sample, in a manner that allowed the reader to examine individual change, in concert with relative standing on several personality characteristics. Such analyses provide for a simpler demonstration of personality–intelligence relations, when compared to complex multivariate modeling. Ultimately, using both approaches is likely to lead to a greater understanding of how these different trait domains relate to one another, and a capability to use such findings for applied settings, for example, such as identifying students at risk for falling behind their peers in academic and intellectual development.

## References

[1] Demetriou A., Spanoudis G., Žebec M.S., Andreou M., Golino H., Kazi S. (2018). Mind-personality relations from childhood to early adulthood. J. Intell..

[2] Howard D., Schickore J., Steinle F. (2006). Lost wanderers in the forest of knowledge: Some thoughts on the discovery-justification distinction. Revisiting Discovery and Justification: Historical and Philosophical Perspectives on the Contest Distinction.

[3] Ackerman P.L., Heggestad E.D. (1997). Intelligence, personality, and interests: Evidence for overlapping traits. Psychol. Bull..

[4] Bayley N. (1968). Behavioral correlates of mental growth: Birth to thirty-six years. Am. Psychol..

